# Multicentre cohort study on the course of paediatric familial Mediterranean fever in the aftermath of the 2023 earthquake

**DOI:** 10.1136/bmjpo-2025-003818

**Published:** 2026-05-04

**Authors:** Veysel Cam, Siddika Songul Yalcin, Ezgi Deniz Batu, Hatice Melisa Kacmaz, Nesibe Gökçe Kocamaz, Özge Günal, Eda Kayhan, Burcu Bozkaya, Semanur Taşkın, Vildan Gungorer, Elif Kılıç Konte, Gulsah Pirim, Dilara Ünal, Semanur Ozdel, Oya Koker, Aysenur Pac Kısaarslan, Rabia Miray Kisla Ekinci, Şeyda Dogantan, Selcan Demir, Kubra Ozturk, Hatice Adıgüzel Dundar, Ozge Basaran, Ayşe Balat, Betul Sozeri, Ozgur Kasapcopur, Yelda Bilginer, Seza Ozen

**Affiliations:** 1 Department of Paediatrics, Division of Rheumatology, Hacettepe University, Ankara, Turkey; 2 Social Paediatrics, Hacettepe University Faculty of Medicine, Ankara, Turkey; 3 Department of Paediatrics, Division of Rheumatology, Gaziantep University Faculty of Medicine, Gaziantep, Turkey; 4 Department of Paediatrics, Division of Rheumatology, Ministry of Health Ankara Etlik City Hospital, Ankara, Turkey; 5 Department of Paediatrics, Marmara University Faculty of Medicine, Istanbul, Turkey; 6 Faculty of Medicine, Department of Paediatrics, Division of Rheumatology, Erciyes University, Kayseri, Turkey; 7 Department of Paediatrics, Division of Rheumatology, TC Sağlık Bakanlığı Samsun Eğitim ve Araştırma Hastanesi, Samsun, Turkey; 8 Department of Paediatrics, Division of Rheumatology, Diyarbakir Egitim ve Arastirma Hastanesi, Diyarbakır, Turkey; 9 Department of Paediatrics, Division of Rheumatology, TC Sağlık Bakanlığı Ankara Gülhane Eğitim ve Araştırma Hastanesi, Ankara, Turkey; 10 Cerrahpaşa Faculty of Medicine, Paediatric Rheumatology, Istanbul University-Cerrahpasa, İstanbul, Turkey; 11 Department of Paediatrics, Division of Rheumatology, Umraniye Training and Research Hospital, Istanbul, Turkey; 12 Department of Paediatrics, Division of Rheumatology, Hacettepe University Faculty of Medicine, Ankara, Turkey; 13 Department of Paediatrics, Division of Rheumatology, Marmara University Faculty of Medicine, Istanbul, Turkey; 14 Department of Paediatrics, Division of Rheumatology, Erciyes Universitesi, Kayseri, Turkey; 15 Department of Paediatrics, Division of Rheumatology, Çukurova Universitesi, Adana, Turkey; 16 Department of Paediatrics, Division of Rheumatology, TC Sağlık Bakanlığ Başakşehir Çam ve Sakura Şehir Hastanesi, İstanbul, Turkey; 17 Facult of Medicine, Department of Paediatrics, Division of Rheumatology, Eskisehir Osmangazi University, Eskişehir, Turkey; 18 Department of Paediatrics, Division of Rheumatology, Istanbul Medeniyet University, Istanbul, Turkey; 19 Department of Paediatrics, Division of Rheumatology, TC Sağlık Bakanlığı SBÜ Dr Behçet Uz Çocuk Hastalıkları Ve Cerrahisi Eğitim Ve Araştırma Hastanesi, Izmir, Turkey; 20 Department of Paediatrics, Division of Rheumatology, Istanbul University-Cerrahpasa Cerrahpasa Faculty of Medicine, Istanbul, Turkey

**Keywords:** Rheumatology

## Abstract

**Objectives:**

Türkiye was struck by earthquakes on 6 February 2023. Emerging evidence links psychosocial stress and environmental insults to inflammasome activation. This multicentre study investigated whether the earthquake was associated with changes in the disease course among paediatric patients with familial Mediterranean fever (FMF).

**Methods:**

Data were collected from 963 paediatric FMF patients (<18 years) across fifteen centres, including three located in the earthquake zone. Monthly attack counts were analysed over an 18-month period (February 2022–August 2023). Analyses were stratified by earthquake exposure and colchicine access (outside the earthquake area; earthquake area with sufficient access; earthquake area with limited access for the first 2 weeks). Primary analyses used multilevel interrupted time-series and difference-in-differences Poisson models for monthly attack counts, and secondary analyses used mixed-effects logistic regression for monthly attack presence (≥1 attack). Both models were stratified by colchicine access and adjusted for clinical covariates.

**Results:**

In the overall cohort, the February 2023 earthquake was associated with a 35% immediate rise in monthly FMF attack frequency (incidence rate ratio (IRR)=1.35, 95% CI 1.15 to 1.59). This effect was markedly amplified within the earthquake zone: attack frequency increased 2.16-fold in regions with adequate colchicine access (IRR=2.16, 95% CI 1.49 to 3.12) and 3.56-fold in regions with limited access (IRR=3.56, 95% CI 2.79 to 4.55). A significant post-earthquake decline in attacks followed (monthly trend IRR=0.93, 95% CI 0.89 to 0.98), with the fastest recovery in limited-access regions (IRR=0.83, 95% CI 0.78 to 0.89). Findings were consistent in the secondary logistic model.

**Conclusion:**

FMF patients in the earthquake zone experienced a marked but transient escalation in disease activity after the disaster, with the strongest impact in regions with limited medication access. These findings suggest that not just access to treatment but also psychosocial stress or possibly environmental factors may cause activation of such diseases through the trigger of pyrin-inflammasome.

**WHAT IS ALREADY KNOWN ON THIS TOPIC**

- Familial Mediterranean fever (FMF) attacks are known to be triggered by several factors, including infections, environmental exposures and psychological stress.

**WHAT THIS STUDY ADDS**

- This study shows that large-scale natural disasters can trigger FMF attacks in a substantial number of children, likely through combined effects of psychosocial stress and environmental disruption.

**HOW THIS STUDY MIGHT AFFECT RESEARCH, PRACTICE OR POLICY**

- These findings support the need for disaster preparedness strategies that ensure continuity of care, equitable access to medication and early psychosocial support for children with chronic autoinflammatory diseases.

## Introduction

 Familial Mediterranean fever (FMF) is a monogenic autoinflammatory disease (AID) characterised by recurrent episodes of febrile serositis.^1^ It is the most common monogenic AID in populations of Eastern Mediterranean origin. The disease is caused by gain-of-function mutations in the *MEFV* gene encoding the pyrin protein.^2^ Overactive pyrin inflammasome leads to uncontrolled secretion of IL-1 β and causes FMF symptoms.^3 4^ FMF attacks can be triggered by infection, trauma, psychological stress, excessive physical activity, exposure to cold and menstruation.^5^ Infections are among the known stimuli of the pyrin inflammasome. Recent data has elegantly shown an association between various forms of steroids and the activation of inflammasomes.^6^ On the other hand, it is known that environmental pollutants such as asbestos, silica and oxidative stress are triggers for different inflammasome structures (eg, *NLRP3, NLRP6*). Moreover, it has been suggested that *NLRP3* polymorphisms may affect the risk of asbestos-related diseases.^7^

The earthquake in the Eastern Mediterranean region of Türkiye on 6 February 2023, affected 11 provinces and 15% of the population in Türkiye, with six of these provinces being primarily affected. The widespread destruction and ensuing chaos became a prolonged source of psychological stress and environmental pollution for survivors. For patients requiring daily medications, such as those with chronic rheumatic or AIDs, access to treatment also became a significant challenge. Only a few studies have examined the course of rheumatic diseases during large-scale natural disasters.^8 9^ However, for an AID associated with a monogenic aetiology causing inflammasome activation and characterised by flares of inflammation, substantial environmental changes may play a key role in modulating disease activity. Immediately after the earthquake, paediatric rheumatologists living in the affected cities of Türkiye reported an increase in FMF flares (unpublished data). Therefore, the present study aimed to evaluate how the earthquake influenced FMF flares and to explore the association between disease activity and post-disaster environmental and healthcare disruptions.

This study offers insight into how environmental changes following an earthquake may influence the activity of monogenic AIDs such as FMF. This may enhance our understanding of the relationship between genetic factors and environmental interactions. The data could be valuable, especially for developing disease management strategies that respond rapidly to environmental changes. The study can guide developing or improving emergency plans for managing individuals with chronic AIDs in similar natural disasters.

## Methods

### Study design and participants

This study employed a mixed interrupted time-series (ITS) and difference-in-differences (DiD) design to evaluate temporal changes in monthly attack counts among children before and after the February 2023 earthquake. Monthly counts were available for each child from February 2022 to August 2023, corresponding to 12 pre-earthquake and six post-earthquake months. Each child contributed repeated measures nested within one of 15 clinical centres.

This multicentre cohort study included paediatric patients (<18 years of age) with a confirmed diagnosis of FMF who had been diagnosed at least 1 year before the 6 February 2023, earthquakes in Türkiye. Patients were recruited from 15 centres across the country, three of which are in the earthquake-affected region. All patients met the EUROFEVER diagnostic criteria for FMF.^10^

Patients diagnosed during or after the earthquake and those lacking confirmatory *MEFV* genotypes according to EUROFEVER criteria were excluded. Likewise, patients receiving biologic therapies were excluded, as the strong effect of biologics may obscure the effects of the earthquake.

### Data collection

Demographic characteristics, clinical features, *MEFV* genotype, medication use and comorbidities were recorded.

The frequency of FMF attacks was assessed for the 18-month period from February 2022 to August 2023, including both the 12 months preceding the earthquake (February 2022–January 2023) and the 6 months following it (February–August 2023). Attack counts were recorded from clinical follow-up documentation and patient/parent self-reports at monthly visits. Only patients with complete and regular follow-up data were included to minimise recall bias.

### Dependent and independent variables

The dependent variables were the monthly number of attacks and monthly attack presence.

The key independent variable was the earthquake period, coded as 0=pre-earthquake (February 2022–January 2023) and 1=post-earthquake (February–August 2023).

Time was represented by three components:

- time—continuous month sequence across the entire study period.
- post—indicator of the post-earthquake period (level change).
- time_after—months elapsed since the earthquake (slope change).

Geographic exposure was defined by region:

- Living outside the earthquake zone: unaffected children.
- Earthquake area—sufficient drug access: areas affected by the earthquake where structural damage was minimal and medication supply remained uninterrupted.
- Earthquake area—limited drug access: areas within the primary impact zone, where more substantial destruction led to temporary interruption in colchicine availability—lasting up to 2 weeks—after which access was restored.

Interaction terms between post and Region012, and between time_after and Region012, captured regional differences in both immediate and longitudinal effects.

Potential confounders included age group (<12/≥12 years), age at diagnosis (months), sex (male/female), *MEFV* genotype and comorbidity/drug-use status (none/comorbidity without medication/comorbidity with medication).

### Sample size and ethical approval

Given an estimated FMF prevalence of 1–3 per thousand in Türkiye, power analysis indicated that a minimum of 654 cases was required to detect an OR of 1.5 between groups with 95% power and a 5% alpha error (G*Power V.3.1.9.4, Kiel, Germany).^11^ Accounting for potential missing data (20%), the planned sample size was 800 patients.

### Statistical analysis

Analyses were performed using SPSS software and R (V.4.4.1). R analyses were conducted using the glmmTMB, emmeans, broom.mixed, performance, DHARMa, dplyr and ggplot2 packages.

Categorical variables were summarised as frequencies (n, %) and continuous variables as mean±SD or median (IQR), as appropriate.

### Modeling attack counts

To assess the temporal and regional changes in monthly attack counts before and after the earthquake, we fitted a multilevel ITS and DiD Poisson mixed-effects regression model. The model was implemented using the glmmTMB package (R V.4.4.1), which allows estimation of both random intercepts and random slopes for longitudinal count data. This approach accounts for within-subject correlation across repeated monthly measures and between-centre variability.

Each individual had up to 18 monthly observations (February 2022–August 2023), nested within 15 healthcare centres. The model incorporated a random intercept for centre to capture clustering by site, a random intercept and random slope for time_after per patient (ID) to account for subject-level heterogeneity in temporal trends. The outcome variable was the monthly attack count (attacks).

Fixed effects included time variables (post_eq: binary indicator (0=pre-earthquake, 1=post-earthquake); time_after: number of months since February 2023 (0 for pre-earthquake)), regional grouping (outside earthquake area, earthquake area with sufficient drug access, earthquake area with limited drug source), interaction terms (region×post_eq and region×time_after to estimate both immediate (level) and trend (slope) differences between groups) and adjustment covariates (current age (<12 vs ≥12 years), sex (female vs male), age at diagnosis (continuous), mutation class (1–5), comorbidity and drug use (0=none, 10=comorbidity without drug, 11=comorbidity with drug use)).

Incidence rate ratios (IRRs) and 95% CIs were derived by exponentiating fixed-effect coefficients. Model diagnostics were performed using DHARMa residual simulation tests for overdispersion and zero inflation, which indicated no model misspecification (dispersion ratio=0.73, p=0.17; zero inflation p=0.23). The final Poisson mixed-effects model demonstrated good fit (AIC (Akaike Information Criterion)=15 946), with hierarchical variance (Random effects) estimates of SD=0.467 for centres, SD=0.816 for individual intercepts and SD=0.106 for the random slope of time_after (correlation=–0.74).

### Modeling attack presence: model specification for the binary outcome (≥1 attack per month)

We modelled the probability of experiencing at least one attack in a given month (binary outcome: attack=1 if ≥1 attack, else 0) between February 2022 and August 2023 to assess both immediate (level) and gradual (slope) post-earthquake effects across regions using a multilevel mixed-effects logistic regression. The specification combined ITS and DiD components.

Three time variables were defined: (1) *time*—continuous monthly trend before the earthquake, (2) *post_eq* —indicator for the post-earthquake period (0=pre–February 2023; 1=February 2023 and later) and (3) *time_after*—number of months since February 2023 (0 before the earthquake).

Regions were categorised as (reference) outside the earthquake area, earthquake area with adequate colchicine access and earthquake area without colchicine access.

The fixed-effects structure included the three time variables, region, their interactions (*region×post_eq* for extra level change and *region×time_after* for post-earthquake slope change), and individual-level covariates: age group (<12 vs ≥12 years), sex (female vs male), age at diagnosis (continuous), mutation class (reference=Exon 10 homozygous) and comorbidity status (*comorbidity present, no regular drug required; comorbidity present, requires continuous medication vs no comorbidity*).

Random intercepts were included for centre (15 sites) and participant (ID; n=963) to account for clustering and repeated monthly observations (18 297 child-months).

The probability of experiencing at least one attack during month *t* for child *i* in centre *j* was modelled as:

Y_ijt_∼Bernoulli(p_ijt_), logit(p_ijt_)=η_ijt_


$$\begin{array}{l} \eta_{ijt}=\alpha_{0}+\alpha_{1}\;{\text{time}}_{t}+\alpha_{2}\;{\text{post}}_{t}+\alpha_{3}\;{time\_after}_{t}\\ \;+\alpha_{4}^{⊤}G_{j}+\alpha_{5}^{⊤}\left({post}_{t}\times G_{j}\right)+\alpha_{6}^{⊤}\left({time\_after}_{t}\times G_{j}\right)\\ \;+\gamma^{⊤}Z_{ij}+u_{0j}+v_{0i(j)} \end{array}$$

where

- Yijt denotes whether child *i* in centre *j* experienced ≥1 attack in month *t* (1=yes, 0=no).
- p_ijt_=P (Y_ijt_=1), and *logit (p*)=log [p/(1−p)].
- *time* represents the continuous monthly trend prior to the earthquake.
- *post* indicates the post-earthquake period (0=February 2022–January 2023, 1=February 2023–August 2023).
- *time_after* captures the monthly change in trend following the earthquake.
- G_j_ encodes the regional group.
- Z_ij_ includes individual-level covariates.
- u_0j_∼N (0, σ_u_^2^) and v_0 i(j)_∼N (0, σ_v_^2^) represent random intercepts for centre and individual, respectively, to account for hierarchical clustering and repeated monthly observations.

Exponentiated fixed-effect coefficients (exp (α_k_)) were interpreted as ORs. Interaction terms between *post* and region capture differences in immediate level change (DiD effects), whereas interactions between *time_after* and region capture differences in post-earthquake trend (slope) changes (ITS effects).

The model was estimated using a binomial distribution with a logit link via the glmmTMB package (R V.4.5). ORs and 95% CIs were derived from exponentiated fixed-effect coefficients. Marginal post-earthquake contrasts and post-earthquake slopes were estimated with emmeans and emtrends, respectively, on the logit scale and back-transformed to ORs.

Model fit indices indicated adequate convergence and variance partitioning (AIC=14 5090.4; random-effect SDs: centre=0.610, participant=1.028).

### Patient and public involvement

The research question and outcome measures were based on routine clinical follow-up practices. Prior to the initiation of the study, monthly attack frequency and related clinical features were already being assessed as part of standard patient management, thereby indirectly reflecting patient priorities in disease monitoring. No patients or members of the public were directly involved in the design, conduct, reporting or dissemination of this study. Study results will be communicated to patients during routine outpatient visits on request.

## Results

**Figure 1:**
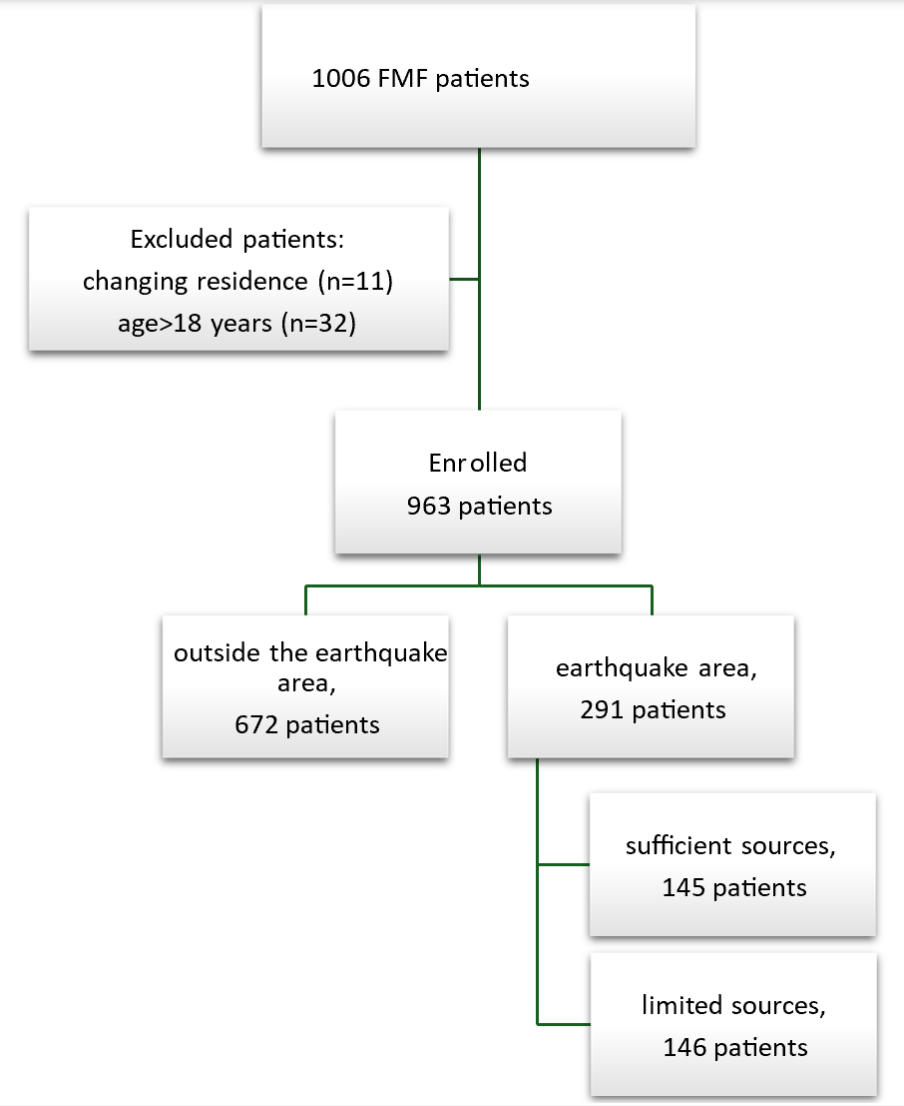
Flow diagram for paediatric patients with FMF. FMF, familial Mediterranean fever.

A total of 1006 FMF patients from fifteen different centres in Türkiye were included in this study. Patients who were in the earthquake area but who left their place of residence within the first week and who were over 18 years of age were excluded (figure 1); finally, 963 paediatric patients were included with a mean age at diagnosis of 11.1±4.0 years11.1±4 (mean±SD) years (table 1). In this cohort, 52.3% were girls. Approximately 80% of patients had exon 10 *MEFV* mutations. The most common comorbidity was juvenile idiopathic arthritis (JIA) followed by immunoglobulin A (IgA) vasculitis (table 1).

**Table 1:** The characteristics of paediatric patients with familial Mediterranean fever (n=963)

Characteristics	%
Age <12 years, n (%)	536 (55.7)
Sex, female, n (%)	504 (52.3)
Age, mean±SD (median), months	132.9±48.5 (132)
Age at diagnosis, mean±SD (median), months	65.6±39.3 (58)
Duration of illness, mean±SD (median), months	67.3±39.1 (60)
Mutation, n (%)	
Exon 10 homozygous	474 (49.2)
M694V	384 (39.9)
M680I	61 (6.3)
V726A	19 (2.0)
M694I	7 (0.7)
R761H	5 (0.5)
Exon 10 compound heterozygous	284 (29.5)
M694V/M680I	108 (11.2)
M694V/V726A	93 (9.7)
M680I/V726A	33 (3.4)
M694V/R761H	23 (2.4)
M694V/M694I	12 (1.2)
Others	15 (1.6)
Non exon 10 homozygous	43 (4.5)
E167D/E167D	16 (1.7)
K695N/K695N	4 (0.4)
F479L/F479L	8 (0.8)
E148Q/E148Q	15 (1.6)
Non exon 10 compound heterozygous	52 (5.4)
F479L/E167D	19 (2.0)
F479L/T267I	3 (0.3)
F479L/K695N	9 (0.9)
E167D/K695N	10 (1.0)
E167D/L396F	5 (0.5)
F479L/E403K	4 (0.4)
E167D/T267I	2 (0.2)
Exon 10/non exon 10 compound heterozygous	110 (11.4)
M694V/F479L	41 (4.3)
M694V/E167D	38 (3.9)
M694V/F479L	8 (0.8)
M680I/E167D	11 (1.1)
V726A/E167D	4 (0.4)
V726A/K695N	5 (0.5)
M680I/K695N	3 (0.3)
Comorbidity, n (%)	123 (12.8)
Juvenile Idiopathic Arthritis	35 (3.6)
IgA Vasculitis	20 (2.1)
IBD	12 (1.2)
Others	65 (6.3)
Comorbidity with regular drug use	61 (6.3)
Region of residence, n (%)	
Earthquake area with limited sources	146 (15.1)
Earthquake area with sufficient sources	145 (15.1)
Outside of earthquake area	672 (69.8)

IBD, Inflammatory Bowel Disease; SD, Standard deviation.

The number of patients from each centre is presented in online supplemental table 1). 291 (30.2%) of the cases were from hospitals in the earthquake area. 146 cases were temporarily unable to access colchicine for 2 weeks after the earthquake.

A total of 963 children from 15 healthcare centres contributed 18 297 monthly observations between February 2022 and August 2023.

### Change in attack counts

**Figure 2:**
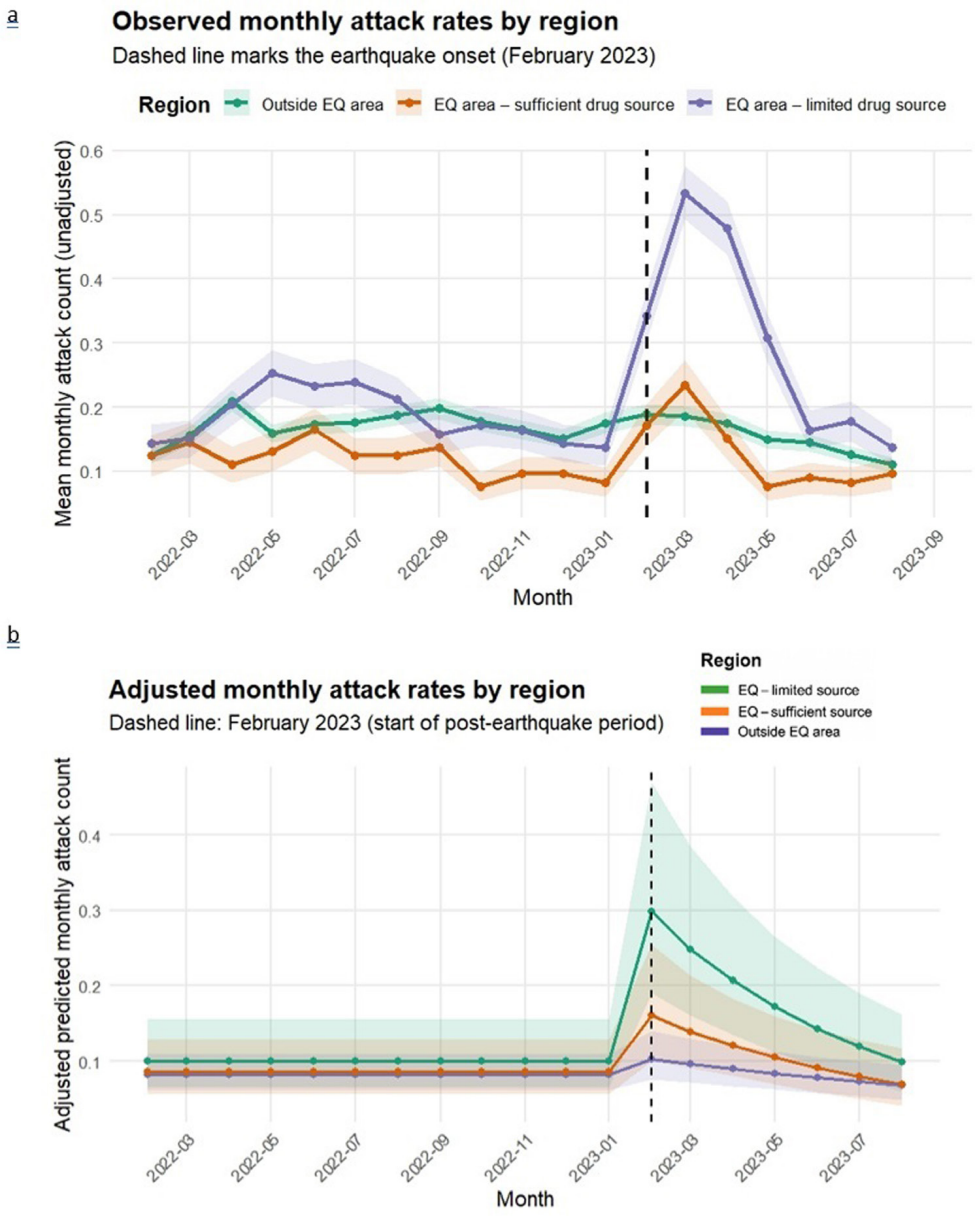
**(a**) Observed monthly attack rates by region and (**b**) adjusted monthly attack rates by region (predicted from the mixed-effects Poisson model).

Figure 2a illustrates the unadjusted temporal pattern of monthly attack counts. Prior to the earthquake, mean attack rates remained low and stable across all regions. A marked surge occurred immediately after the earthquake in February 2023, with the most pronounced increase observed in centres located within the earthquake zone where colchicine was unavailable. Rates subsequently decreased in all groups, approaching pre-earthquake levels by mid-2023. This pattern visually supports the model-based findings, showing a sharp transient escalation in attacks following the earthquake and a progressive normalisation over subsequent months.

The multilevel Poisson mixed-effects regression model identified significant temporal and regional differences in monthly attack rates following the earthquake (table 2). The overall post-earthquake level change (February 2023 vs January 2023) indicated a 35% increase in attack counts (IRR=1.35; 95% CI 1.15 to 1.59; p<0.01). This immediate rise was followed by a steady decline over time, as reflected by the monthly post-earthquake trend (IRR=0.93; 95% CI 0.89 to 0.98; p<0.01), corresponding to an average 7% decrease per month during February–August 2023. At baseline (pre-earthquake), regional differences between the earthquake-affected and outside areas were not statistically significant (sufficient vs outside: IRR=1.04; p=0.82; limited vs outside: IRR=1.23; p=0.30). However, the interaction effects revealed substantial heterogeneity in post-earthquake responses. Relative to the outside region, attack rates increased 1.6-fold in the sufficient drug-source region (IRR=1.60; 95% CI 1.07 to 2.39; p=0.02) and 2.6-fold in the limited drug access region (IRR=2.64; 95% CI 1.97 to 3.53; p<0.01) immediately after the earthquake. Post-earthquake trends also varied by region. The sufficient access area exhibited a mild and non-significant monthly decline (IRR=0.93; 95% CI 0.84 to 1.03; p=0.15), whereas the limited access area showed a significantly faster downward trend (IRR=0.89; 95% CI 0.83 to 0.96; p*<*0.01), indicating a sharper recovery trajectory compared with other regions. Among covariates, the presence of comorbidity without drug treatment (IRR=1.33; 95% CI 1.05 to 1.68; p=0.02) was significantly associated with higher attack rates. Age group (≥12 years) and age at diagnosis did not show significant associations with attack frequency.

**Table 2:** Interrupted time-series+difference-in-differences mixed Poisson model (IRR, 95% CI)

Term	IRR	95% CI	P value
Post (level change, February 2023)	**1.35**	1.15 to 1.59	0.0003
Post-trend (time_after, per month)	**0.93**	0.89 to 0.98	0.002
Earthquake area – colchicine_available versus outside (baseline)	1.04	0.73 to 1.50	0.820
Earthquake area – colchicine_unavailable versus outside (baseline)	1.23	0.83 to 1.81	0.300
Post×colchicine_available (extra level change)	**1.60**	1.07 to 2.39	0.022
Post×colchicine_unavailable (extra level change)	**2.64**	1.97 to 3.53	<0.001
Post-trend×colchicine_available (extra monthly change)	0.93	0.84 to 1.03	0.150
Post-trend×colchicine_unavailable (extra monthly change)	**0.89**	0.83 to 0.96	0.002
Age ≥12 years (vs <12 years)	1.09	0.94 to 1.26	0.270
Age at diagnosis (per year)	0.97	0.90 to 1.04	0.410
Sex (female vs male)	**1.17**	1.04 to 1.33	0.012
Comorbidity_no_drug (vs none)	**1.33**	1.05 to 1.68	0.020
Comorbidity_with_drug use (vs none)	1.24	0.97 to 1.58	0.086
Mutation class 2 (vs 1)	**1.18**	1.02 to 1.37	0.030
Mutation class 3 (vs 1)	1.00	0.73 to 1.39	0.980
Mutation class 4 (vs 1)	0.78	0.58 to 1.04	0.092
Mutation class 5 (vs 1)	1.00	0.81 to 1.23	1.00

Model fit and random effects: AIC=15 946; random-intercept SD (centre)=0.47, SD (ID)=0.82; random-slope (time_after) SD=0.106, corr=−0.74. Dispersion test p=0.17 (no over-dispersion); zero-inflation p=0.23.

Statistically significant IRR values are shown in bold (p < 0.05).

IRR, incidence rate ratio.

When the immediate level change (February 2023) was evaluated relative to the pre-earthquake month (January 2023), the outside-area group showed a 35% increase in monthly attack counts (IRR=1.35, 95% CI 1.15 to 1.59; p<0.01). The increase was substantially greater in the earthquake-affected regions: 2.16-fold in areas with sufficient drug sources (95% CI 1.49 to 3.12; p<0.01) and 3.56-fold in areas with limited drug sources (95% CI 2.79 to 4.55; p<0.01) (table 3).

**Table 3:** Immediate level change at the earthquake (February 2023): post versus pre within each group (emmeans), and post-earthquake monthly trend (February–August 2023): IRR per month (emtrends)

Group	Immediate level change (Post/Pre) IRR (95% CI)	P value	Slope β (log)	IRR per month (95% CI)	P value
Outside earthquake area	**1.35 (1.15 to 1.59)**	<0.001	–0.069	**0.93 (0.89 to 0.98)**	0.002
Earthquake area – **colchicine_available**	**2.16 (1.49 to 3.12)**	<0.001	–0.141	**0.87 (0.79 to 0.95)**	0.003
Earthquake area – **colchicine_unavailable**	**3.56 (2.79 to 4.55)**	<0.001	–0.184	**0.83 (0.78 to 0.89)**	<0.001

Statistically significant IRR values are shown in bold (p < 0.05).

* Adjusted for age group (<12 vs ≥12 years), age at diagnosis, sex, mutation class and comorbidity/drug status; evaluated at time=February 2023 (time_after=0).

† Pairwise slope differences (Tukey-adjusted p): Outside versus Sufficient Δβ=+0.071 (p=0.33, ns); Outside versus Limited Δβ=+0.114 (p=0.005); Limited versus Sufficient Δβ=+0.043 (p=0.72, ns).

IRR, incidence rate ratio.

Post-earthquake trends (February–August 2023) demonstrated a consistent decline in monthly attack counts across all regions. The modelled IRR per month was 0.93 for the outside area (≈7% decrease per month; p=0.002), 0.87 for the sufficient-source area (≈13% decrease; p=0.0), and 0.83 for the limited-source area (≈17% decrease; p<0.01). The downward trend was significantly steeper in the limited-source region compared with the outside area (Δβ=0.114; p=0.005), whereas differences between the sufficient-source and other regions were not statistically significant (table 3).

Figure 2b adjusted monthly attack rates by region (predicted from the mixed-effects Poisson model). The dashed line marks February 2023, the onset of the post-earthquake period. A sharp immediate surge in attack rates is observed at the time of the earthquake, particularly in centres without colchicine access, followed by a progressive decline over subsequent months. By August 2023, attack frequencies had approached pre-earthquake levels across all regions, with the most rapid recovery seen in the colchicine-unavailable group.

### Change in attack presence

Using the mixed-effects logistic regression model, a significant immediate increase was observed in the odds of experiencing at least one attack following the February 2023 earthquake, with substantial regional heterogeneity (table 4). Compared with the pre-earthquake period, the odds increased by 40% outside the earthquake zone (OR=1.40; 95% CI 1.13 to 1.74; p=0.002). The rise was far greater within the affected regions: 1.9-fold in areas with adequate colchicine access (OR=1.89; 95% CI 1.16 to 3.10; p=0.01) and 7.0-fold where colchicine access was limited (OR=7.02; 95% CI 4.68 to 10.5; p<0.01).

**Table 4:** Mixed-effects logistic regression (binary outcome: ≥1 attack per month)

Predictor	OR	95% CI	P value
Time (monthly trend, pre-earthquake)	0.997	0.981–1.01	0.667
Post (level change after February 2023)	**1.40**	**1.13–1.74**	**0.002**
Time after (slope change post-earthquake)	**0.895**	**0.854–0.938**	**<0.001**
Earthquake area – colchicine_available	1.04	0.66–1.64	0.878
Earthquake area – colchicine_unavailable	1.21	0.73–2.00	0.453
Age ≥12 years (vs <12 years)	1.09	0.89–1.33	0.407
Age at diagnosis (per year)	0.944	0.853–1.04	0.264
Female sex (vs male)	**1.24**	**1.05–1.47**	**0.011**
Mutation class 2 (vs 1)	1.22	1.00–1.49	0.052
Mutation class 3 (vs 1)	0.962	0.619–1.49	0.863
Mutation class 4 (vs 1)	0.722	0.485–1.08	0.109
Mutation class 5 (vs 1)	1.07	0.803–1.42	0.648
Comorbidity_no_drug (vs none)	**1.54**	**1.11–2.15**	**0.0098**
Comorbidity_with_drug use (vs none)	1.29	0.917–1.81	0.144
Post×colchicine_available	**1.89**	**1.16–3.10**	**0.011**
Post×colchicine_unavailable	**7.02**	**4.68–10.5**	**<0.001**
Time after×colchicine_available	0.899	0.802–1.01	0.069
Time after×colchicine_unavailable	**0.749**	**0.682–0.822**	**<0.001**

Model includes random intercepts for centre (n=15) and participant (n=963); 18 297 child-months. Reference category=outside earthquake area. OR >1 indicates higher odds of at least one attack. Bold indicates statistical significance at p<0.05.

Comorbidity_no_drug: comorbidity present, no regular drug required.

Comorbidity_with_drug: comorbidity present, requires continuous medication.

Among covariates, female sex (OR=1.24; 95% CI 1.05 to 1.47; p=0.01) and comorbidity without regular drug treatment (OR=1.54; 95% CI 1.11 to 2.15; p=0.01) were associated with higher odds of attacks, whereas age, age at diagnosis, and mutation class were not statistically significant (table 4). Within-region contrasts confirmed these findings: attack odds increased 2.66-fold (95% CI 1.68 to 4.22) in the adequate-access group and 9.86-fold (95% CI 6.85 to 14.19) in the limited-access group, versus 1.40-fold outside the affected area (table 5).

**Table 5:** Post-earthquake contrasts (level change) and post-earthquake trends (slope change)

Region	Post-earthquake contrasts (level change)		Post-earthquake trends (slope change)*		
	Post/Pre OR (95% CI)	P value	Time-after β (logit)	OR per month (95% CI)	P value
Outside	**1.40** (1.13 to 1.74)	**0.002**	−0.111	**0.90** (0.85 to 0.94)	**<0.001**
Earthquake area-colchicine_available	**2.66** (1.68 to 4.22)	**<0.001**	−0.217	**0.81** (0.72 to 0.90)	**<0.001**
Earthquake area- colchicine_unavailable	**9.86** (6.85 to 14.19)	**<0.001**	−0.400	**0.67** (0.62 to 0.73)	**<0.001**

Pairwise slope differences (Tukey-adjusted): outside versus adequate p=0.164; outside versus limited p<0.0001; adequate versus limited p=0.020.

Statistically significant OR values are shown in bold (p < 0.05).

During the post-earthquake period, a significant downward trend in monthly attack probability was evident across all regions. The decline was strongest in the limited-access group (β=−0.400; OR=0.67 per month; p<0.01), followed by the adequate-access group (β=−0.217; OR=0.80; p<0.01) and the outside region (β=−0.111; OR=0.90; p<0.01). Tukey-adjusted comparisons showed a significantly steeper decline in the limited-access region compared with both outside (p<0.01) and adequate-access regions (p=0.02, table 5).

## Discussion

In this multicentre cohort of 963 paediatric patients with FMF, we observed a marked increase in attack frequency following the February 2023 earthquake in Türkiye. The increase was most prominent in children living in the primary impact area, where structural destruction was most severe. Although colchicine supply issues in this region were resolved within a maximum of 2 weeks, disease activity continued to rise for a considerably longer period. However, when we assessed patients with adequate colchicine supply we still observed an increase in attacks. A milder but still notable increase occurred among children even outside the directly affected region, suggesting that broader psychosocial or environmental stressors may also have contributed to disease exacerbation.

There are a limited number of studies on how natural disasters affect rheumatological diseases.^8 9^ These studies are often related to autoimmune diseases. In a study investigating the incidence of antineutrophil cytoplasmic antibody (ANCA)-associated vasculitis (AAV), a similar incidence was observed before and after the earthquake in a 7-year follow-up.^12^ In 1999, clinical changes in systemic lupus erythematosus (SLE) patients were investigated after a magnitude seven earthquake in Taiwan.^9^ The cohort consisted of nine patients, and no clinical deterioration was observed. There were no striking changes in the studies evaluating the incidence of AAV and severity of SLE after the earthquake.^9 12^ Unlike autoimmune diseases, which are chronic and slow processes, the relationship between triggers and FMF can be observed over a shorter time interval.

While the temporal association between the earthquake and increased disease activity is striking, caution is warranted in attributing causality. Various overlapping factors—such as psychological stress, environmental pollutants, infections and changes in access to medication—may have contributed to the observed patterns.^5^ Although temporary interruptions in colchicine availability affected only a subset of patients, the increase in attack frequency was also evident among those with continued access, suggesting the involvement of additional mechanisms.

Several known triggers of FMF attacks—including infections, stress, cold exposure and environmental agents—may act synergistically.^13^ In this context, psychological stress emerges as a plausible factor, supported by prior studies linking emotional stress to increased disease activity in autoinflammatory conditions. Moreover, exposure to environmental pollutants such as asbestos and silica—potentially released from damaged infrastructure—may have contributed, as both are known activators of the NLRP3 inflammasome.^14^ However, a direct link between these agents and the pyrin inflammasome has yet to be established. We were unable to test the level of environmental pollutants right after the earthquake, although they may have been contributing factors to inflammation.

Interestingly, attack frequency appeared to normalise approximately 3–5 months after the earthquake, possibly reflecting adaptive mechanisms or the stabilisation of external stressors. Although seasonal variability was considered, the observed increase was not consistent with climatic trends, as temperatures during the same period in the previous year were comparable.^15^ Additionally, both males and females in the earthquake zone experienced increased attack rates, and the impact was similar across different age groups. The role of infections cannot be excluded, especially considering data from previous studies showing increased FMF activity during viral illnesses. Infection history was not systematically captured in this study; however, a marked increase has not been reported in the region.

The magnitude of destruction and early observations by local rheumatologists raised the hypothesis that psychological stress may have been a key contributing factor. Emotional distress may not have been limited to those directly affected by the earthquake but may also have extended to individuals with prior earthquake experiences or relatives in the affected area. In vitro data suggesting that steroid catabolites can trigger the pyrin inflammasome—particularly in patients with exon 10 mutations—may provide mechanistic support for a stress-related flare response.^6^ In our cohort, in which approximately 80% of patients carried exon 10 mutations, the pattern of increased attacks followed by normalisation supports this hypothesis, although it remains speculative.

Children with comorbid conditions but without pharmacological treatment had higher attack frequencies, potentially reflecting poorer adherence, limited treatment options or gaps in integrated management of coexisting diseases. This emphasises the need for targeted follow-up and adherence support among children with complex medical needs, particularly in resource-limited settings.

The steeper downward trend in areas with limited access to medications implies that timely access to medications and continuity of care play a key role in mitigating prolonged disease exacerbations following disasters. These findings also reflect differences in physiological stress response, treatment adherence or healthcare utilisation and suggest that targeted follow-up is needed for children with complex medical needs during crisis recovery.

Overall, these findings highlight that the impact of disasters on chronic disease activity is not uniform, but is shaped by baseline socioeconomic and healthcare inequalities. Strengthening medication supply chains, ensuring equitable healthcare access, and establishing continuity-of-care protocols during emergencies should therefore be integral components of paediatric disaster preparedness and resilience planning.

### Strengths and limitations

This study has certain limitations. Although the pre-earthquake period relied on retrospective hospital records, data collection after April 2023 was conducted prospectively, allowing for standardised monthly follow-up and more reliable ascertainment of attack frequency. While colchicine access was largely restored during the follow-up period, data on psychosocial stressors were not systematically collected, preventing assessment of their potential contribution to post-earthquake disease activity. Environmental exposure data (eg, particulate matter, asbestos, silica) were also unavailable, precluding direct evaluation of environmental triggers.

Post-disaster maternal stress and changes in caregiving behaviours have been shown to negatively impact child care practices, including breastfeeding, as evidenced by a recent study conducted after the 2023 Türkiye earthquake.^16^ Parental overprotectiveness, already elevated due to the child’s chronic illness, may be modified by disaster-related stress, contributing to changes in disease activity.^17^ Moreover, parental psychosocial disruptions may be associated with differences in children with chronic conditions.^18^ Additionally, infection history was not systematically assessed, which makes interpretations about potential triggers somewhat speculative. Nevertheless, our study underscores the importance of considering environmental and psychosocial stressors in the management of AIDs—particularly in the aftermath of natural disasters. Furthermore, the 6 month post-earthquake observation period captures only the acute and early recovery phases; longer-term monitoring is needed to determine whether relapse or delayed effects occur. Finally, although diagnostic criteria and reporting formats were standardised across centres, potential differences in healthcare infrastructure or socioeconomic context across sites may still have influenced outcomes. However, our analytic approach incorporated random intercepts for both centre and participant, effectively accounting for hierarchical clustering and between-centre variability. This modelling strategy minimises bias arising from unmeasured site-level differences and strengthens the robustness of our regional comparisons.

To our knowledge, this is one of the first studies to explore the relationship between a large-scale environmental disaster and disease activity in a monogenic autoinflammatory condition. The findings suggest a multifactorial interplay of stress-related and environmental triggers that may influence FMF flares. Further prospective studies incorporating biomarkers and validated stress measures are warranted to better understand these complex interactions.

## Conclusion

In this study, we found that FMF attacks increased in children after the 2023 earthquake, especially in those living in the affected areas. This suggests that stress, environmental changes, and possibly other unknown factors may trigger more flares in this monogenic AID. In subsequent months, attack frequencies declined progressively, indicating gradual adaptation and the re-establishment of medical and social routines. Future disaster prevention plans should therefore prioritise equitable drug distribution, continuity of care for children with chronic conditions, and early psychosocial support to accelerate the return to pre-disaster health trajectories.

## Supplementary material

10.1136/bmjpo-2025-003818 online supplemental file 1

## Data Availability

Data are available upon reasonable request.
