# Supplementary material for: Multicentre cohort study on the course of paediatric familial Mediterranean fever in the aftermath of the 2023 earthquake
Source: BMJ Paediatr Open. 2026 May 4;10(1):e003818. doi: 10.1136/bmjpo-2025-003818 (PMC13141018; doi:10.1136/bmjpo-2025-003818)
Supplement: online supplemental file 1 [file bmjpo-10-1-s001.docx]

**A Multicenter Cohort Study on the course of Pediatric Familial Mediterranean Fever in the Aftermath of the 2023 Earthquake**

**Supplementary Table 1. Number of familial Mediterranean fever (FMF) patients from different pediatric rheumatology centers in Turkey (n=963)** *

| **Name of the center** | **Province** | **FMF patients, n**  **(%)** |
| --- | --- | --- |
| Adana Çukurova University Faculty of Medicine | Adana | 47 (5.0) |
| Ankara City Hospital | Ankara | 50 (5.1) |
| Ankara Etlik City Hospital | Ankara | 113 (11.7) |
| Behçet Uz Pediatric Diseases And Surgery Education Training  And Research Hospital | İzmir | 14 (1.5) |
| Diyarbakır Pediatric Hospital | Diyarbakır | 46 (4.7) |
| Erciyes University Faculty of Medicine | Kayseri | 85 (8.9) |
| Eskişehir Osmangazi University Faculty of Medicine | Eskişehir | 30 (3.1) |
| Gaziantep University Faculty of Medicine | Gaziantep | 200 (20.7) |
| Hacettepe University Faculty of Medicine | Ankara | 78 (8.1) |
| Istanbul Medeniyet University Göztepe Prof. Dr. Süleyman  Yalçın City Hospital | İstanbul | 20 (2.1) |
| Istanbul University Cerrahpasa Faculty of Medicine | İstanbul | 49 (5.1) |
| Marmara University Pendik Training and Research Hospital | İstanbul | 102 (10.6) |
| Mersin City Hospital | Mersin | 32 (3.3) |
| Samsun Training and Research Hospital | Samsun | 64 (6.7) |
| Umraniye Research and Training Hospital | İstanbul | 33 (3.4) |

* Centers located in the earthquake-affected region also monitored patients from non-earthquake areas, and conversely, centers outside the earthquake zone followed patients from the affected regions.
