# Supplementary material for: Multicentre cohort study on the course of paediatric familial Mediterranean fever in the aftermath of the 2023 earthquake
Source: BMJ Paediatr Open. 2026 May 4;10(1):e003818. doi: 10.1136/bmjpo-2025-003818 (PMC13141018; doi:10.1136/bmjpo-2025-003818)
Supplement: Supplementary file 2 [file bmjpo-2025-003818.reviewer_comments.pdf]

PEER REVIEW HISTORY

BMJ Paediatrics Open publishes all reviews undertaken for accepted manuscripts. Reviewers are asked to complete a checklist review form and are provided with free text boxes to elaborate on their assessment. These free text comments are reproduced below.

ARTICLE DETAILS

**TITLE (PROVISIONAL)**	A Multicenter Cohort Study on the course of Paediatric Familial Mediterranean Fever in the Aftermath of the 2023 Earthquake
**AUTHORS**	Cam, Veysel<br>Yalcin, Siddika Songul<br>Batu, Ezgi Deniz<br>Kacmaz, Hatice Melisa<br>Kocamaz, Nesibe Gökçe<br>Günal, Özge<br>Kayhan, Eda<br>Bozkaya, Burcu<br>Taşkın, Semanur<br>Güngörer, Vildan<br>Kılıç Konte, Elif<br>Pirim, Gulsah<br>Ünal, Dilara<br>Ozdel, Semanur<br>Koker, Oya<br>Pac Kısaarslan, Aysenur<br>Kisla Ekinci, Rabia Miray<br>Doğantan, Şeyda<br>Demir, Selcan<br>Ozturk, Kubra<br>Adıgüzel Dündar, Hatice<br>Basaran, Ozge<br>Sozeri, Betul<br>Kasapcopur, Ozgur<br>Bilginer, Yelda<br>Ozen, Seza

VERSION 1 – REVIEW

**REVIEWER NAME**	Flom, Peter
**REVIEWER AFFILIATION**	Peter Flom Consulting
**REVIEWER CONFLICT OF INTEREST**	No Conflict of Interest
**AI DISCLOSURE**	No, I have not used AI
**DATE REVIEW RETURNED**	12-Jul-2025

**GENERAL COMMENTS**	I confine my remarks to statistical aspects of this paper. Unfortunately, there were quite a few problems and I think the analysis has to be redone.<br><br>The biggest issue is that no account was taken of the fact that there are multiple centers. This violates the assumption of independent errors and must be dealt with. One way to do this is with a multilevel model.<br><br>Another issue is using attacks as a yes/no variable. Instead, it

	should be treated as a count variable, and a count method of regression should be used (e.g. Poisson or negative binomila0><br><br>Going page by page:<br><br>p. 4 (abstract) Please give measures of effect size.<br><br>p. 6 please list all the variables and operationalize them. That is, they should be described in enough detail that some other reserarchers could duplicate the work.<br><br>p. 7 I don't see how including only patients with full follow up data helps reduce self report bias.<br><br>The range of FMF should be given with one decimal and some attempt should be made to explain the huge range.<br><br>Rather than earthquake area vs. not, it would be better to use some more sensitive measure. Perhaps distance from the epicenter, or perhaps some other measure of how affected the region was.<br><br>Age should not be dichotomized. Dichotomizing variales is almost always a mistake.<br><br>p. 8 Here you mention a new level "earthquake area with limited resources". Does this variable have two levels or three? Again, probably better to use a continuous measure of how affected the region was by earthquakes, and another of how limited the resources were (surely this varied and did not have only two levels).<br><br>Figure 2 The green line is not labeled. Also, a better graph would be of the difference between one month and the same month a year earlier.

**REVIEWER NAME**	**Pilania, Rakesh**
**REVIEWER AFFILIATION**	Post Graduate Institute of Medical Education and Research (PGIMER), Department of Pediatrics
**REVIEWER CONFLICT OF INTEREST**	No Conflict of Interest
**AI DISCLOSURE**	No, I have not used AI
**DATE REVIEW RETURNED**	22-Sep-2025

**GENERAL COMMENTS**	I read with great interest this study on the impact of natural disasters on the frequency of attacks in FMF patients. The manuscript is trying to address the pertinent topic. However, there are several concerns that need to be addressed.<br>Major Comments:<br>1. Exclusion Criteria on Biologics: The rationale for excluding patients on biologics needs clarification. Patients without biologics may also experience disease flares, so this criterion requires justification.<br>2. Medication Access: It is unclear how patients with only transient difficulties (e.g., during the first two weeks) in accessing medication were categorized as part of the "limited earthquake zone." This needs clearer explanation.<br>3. Results Presentation:<br>o The results are written in overly interpretive language. They should be rewritten for clarity, with factual values provided rather

	than interpretations.<br>o For example, attack frequency was assessed during the one year before the earthquake (February 2022–January 2023) and six months after the earthquake (February–August 2023). However, detailed values are needed to support the findings.<br>o It is important to highlight whether observed differences were clinically as well as statistically significant.<br>4. Recall Bias: Since attack frequency was self-reported, recall bias should be more explicitly acknowledged. Patients with missing data on attack frequency were excluded, but the implications of this exclusion should be discussed.<br>5. Genetic Findings: Approximately 80% of patients had exon 10 MEFV mutations. Variant details should be provided (perhaps in a supplementary file) to support the pathogenicity of the variants.<br>6. Comorbidities: The most common comorbidity was juvenile idiopathic arthritis (JIA), followed by immunoglobulin A (IgA) vasculitis (Table 1). Percentages should be presented alongside actual numbers in the table for better interpretation.<br>7. Residence Data: The percentages of regions of residence presented in Table 1 are confusing and need clarification.<br>8. Study Flow: The flow of patients is unclear. At the start, 1006 pediatric patients are mentioned, but 32 were excluded because they were adults. This creates confusion and should be clarified.<br>9. Figures and Tables:<br>o Figure 2: There are three lines, but color coding is given for only two. This should be corrected.<br>o Supplementary Table 2: The main difference appears between patients in the earthquake area without resources and those outside the earthquake area or with sufficient resources. This suggests multiple reasons for increased episodes (e.g., medication issues). The handling of such confounding factors should be clarified.<br>o Definition of Limited Resources: The manuscript refers to “limited resources,” but this definition is missing. It should be explicitly stated.<br>10. Interpretation of Results:<br>• Results should be presented with actual values before moving to interpretation.<br>• For example, the statement that “patients living in the earthquake zone experienced more frequent flares during the month of the earthquake and the following three months” is valuable, but precise numbers should be shown to confirm the extent of the increase.<br>• The conclusion that patients outside the directly affected area also had increased flares may suggest broader psychosocial or environmental stressors, which should be better substantiated.

VERSION 1 – AUTHOR RESPONSE

Comments from Peter Flom (Reviewer 1)	Answers
I confine my remarks to statistical aspects of this paper. Unfortunately, there were quite a few problems and I think the analysis has to be redone.	We greatly appreciate the reviewer’s insightful comments, particularly the important point about accounting for clustering by center and the recommendation to model the attack variable as a count outcome.
The biggest issue is that no account was taken of the fact that there are multiple centers. This violates the assumption of independent errors and must be dealt with. One way to do this is with a multilevel model.	We fully agree that the presence of multiple centers violates the independence assumption, the multicenter structure of the study may introduce clustering, and therefore the assumption of independence of observations may be violated. We have therefore reanalyzed the data using Poisson multilevel mixed-effects regression model

Another issue is using attacks as a yes/no variable. Instead, it should be treated as a count variable, and a count method of regression should be used (e.g. Poisson or negative binomila0>	with random intercepts for both center and individual to properly account for within-cluster correlation and repeated monthly measures.<br>As your recomendation, in the initial analysis, we compared the attack frequency using Poisson regression models depending on the degree of dispersion observed in the data. The multilevel structure will be retained in these models to address clustering by center.<br>We also acknowledge the concern regarding modeling attack frequency as a binary (yes/no) outcome. This simplification was initially chosen because patient- and parent-reported monthly attack data are prone to variable precision. We modelled the probability of experiencing at least one attack in a given month (binary outcome: attack = 1 if ≥1 attack, else 0) between February 2022 and August 2023 to assess both immediate (level) and gradual (slope) post-earthquake effects across regions using a multilevel mixed-effects logistic regression. The specification combined interrupted time-series (ITS) and difference-in-differences (DiD) components.
Going page by page:<br>p. 4 (abstract) Please give measures of effect size.	Results section of abstract was revised as ": In the overall cohort, the February 2023 earthquake was associated with a 35% immediate rise in monthly FMF attack frequency (IRR = 1.35, 95% CI 1.15–1.59). This effect was markedly amplified within the earthquake zone: attack frequency increased 2.16-fold in regions with adequate colchicine access (IRR = 2.16, 95% CI 1.49–3.12) and 3.56-fold in regions with limited access (IRR = 3.56, 95% CI 2.79–4.55). A significant post-earthquake decline in attacks followed (monthly trend IRR = 0.93, 95% CI 0.89–0.98), with the fastest recovery in limited-access regions (IRR = 0.83, 95% CI 0.78–0.89). Findings were consistent in the secondary logistic model.<br>"
p. 6 please list all the variables and operationalize them. That is, they should be described in enough detail that some other reserarchers could duplicate the work.	Was added.
p. 7 I don't see how including only patients with full follow up data helps reduce self report bias.	"This multicenter cohort study included pediatric patients (<18 years of age) with a confirmed diagnosis of familial Mediterranean fever (FMF) who had been diagnosed at least one year before the February 6, 2023 earthquakes in Türkiye." "Attack counts were recorded from clinical follow-up documentation and patient/parent self-reports at monthly visits. Only patients with complete and regular follow-up data were included to minimize recall bias. "
The range of FMF should be given with one decimal and some attempt should be made to explain the huge range.	Thank you for this valuable comment. We have now presented the range of FMF duration with one decimal point for consistency.
Rather than earthquake area vs. not, it would be better to use some more sensitive measure. Perhaps distance from the epicenter, or perhaps some other measure of how affected the region was.	We appreciate this insightful suggestion. The idea of using a continuous measure such as distance from the epicenter is excellent and would provide more granularity. However, detailed geographical exposure data were not available at the individual level due to privacy constraints and multi-center data collection design. Instead, we used a pragmatic categorical classification based on both geographic location and verified post-disaster healthcare access status: (1) outside earthquake zone, (2) within earthquake

	zone with sufficient access to medical resources, and (3) within earthquake zone with limited access. We have emphasized this limitation and clarified the rationale for this categorization in the Methods and Discussion sections.
Age should not be dichotomized. Dichotomizing variales is almost always a mistake.	We fully agree with the reviewer that dichotomization of continuous variables may lead to loss of information. In the initial analyses, we followed the age at study inclusion based clinical grouping commonly used in pediatric rheumatology (<12 vs. ≥12 years) to align with developmental and treatment-related differences. Following the reviewer's advice, we have now repeated the main analyses using *age at diagnosis as a continuous variable* (in months). The results remained consistent, confirming the robustness of the findings. We have updated the Methods and Tables accordingly.
p. 8 Here you mention a new level "earthquake area with limited resources". Does this variable have two levels or three? Again, probably better to use a continuous measure of how affected the region was by earthquakes, and another of how limited the resources were (surely this varied and did not have only two levels).	Thank you for requesting this clarification. The variable "Region" indeed includes three levels:<br>(0) Outside earthquake zone,<br>(1) Earthquake zone – adequate access to medical resources, and<br>(2) Earthquake zone – limited access to medical resources.<br>This classification was based on hospital-reported operational capacity and medication supply status confirmed during the post-earthquake period.<br>"Earthquake area with limited/no drug access: earthquake-affected provinces where medication shortages or delivery problems for the first two weeks after the event, however, they got their colchicine before 3rd week."
Figure 2 The green line is not labeled. Also, a better graph would be of the difference between one month and the same month a year earlier.	The missing label for the green line has been corrected, and the figure legend has been revised for clarity. Following the reviewer's helpful suggestion, we have also generated an additional figure showing the *monthly difference in attack rates compared to the same month of the previous year* to better illustrate the temporal change.
Sorry to be so negative	On the contrary, we are very grateful for the reviewer's detailed and constructive critique. Each of the points raised has contributed meaningfully to improving the clarity, transparency, and methodological robustness of our manuscript. We appreciate the time and expertise dedicated to reviewing our work.

Comments from Dr. Rakesh Pilania (Reviewer 2)	Answers
I read with great interest this study on the impact of natural disasters on the frequency of attacks in FMF patients. The manuscript is trying to address the pertinent topic. However, there are several concerns that need to be addressed.	We would like to sincerely thank the reviewer for their thoughtful and constructive comments. We have carefully revised the manuscript to address each concern. The changes have substantially improved the clarity and scientific rigor of the paper. Below we provide detailed point-by-point responses.
Major Comments:<br>1. Exclusion Criteria on Biologics: The rationale for excluding patients on biologics needs clarification. Patients without biologics may also experience disease flares, so this criterion requires justification.	Patients receiving biologic therapy were excluded because biologics (especially IL-1 inhibitors such as anakinra or canakinumab) have a major suppressive effect on inflammasome-mediated activity and attack frequency. Including them would therefore bias comparisons of natural disease activity before and after the earthquake. Thus we have included in the text that biologics

	might have obscured the effect of earthquake on basal innate immune system activation.
2. Medication Access: It is unclear how patients with only transient difficulties (e.g., during the first two weeks) in accessing medication were categorized as part of the "limited earthquake zone." This needs clearer explanation.	Thank you for pointing this out. "Limited access" was defined as residing in one of the six provinces within the primary impact zone, where colchicine availability was temporarily interrupted—lasting up to two weeks—following the earthquake. This definition has now been explicitly added to the Methods section: ""Earthquake area – sufficient drug access: Areas affected by the earthquake where structural damage was minimal and medication supply remained uninterrupted.<br><br>Earthquake area – limited drug access:<br>Areas within the primary impact zone, where more substantial destruction led to temporary interruption in colchicine availability—lasting up to two weeks—after which access was restored.."
3. Results Presentation:<br>o The results are written in overly interpretive language. They should be rewritten for clarity, with factual values provided rather than interpretations.<br>o For example, attack frequency was assessed during the one year before the earthquake (February 2022–January 2023) and six months after the earthquake (February–August 2023). However, detailed values are needed to support the findings.<br>o It is important to highlight whether observed differences were clinically as well as statistically significant.	We have fully rewritten the Results section to present exact effect estimates and 95% confidence intervals. Quantitative results are now clearly reported before interpretation, for example: "In the overall cohort, the incidence rate ratio (IRR) for monthly attack frequency increased by 27% immediately after the earthquake (IRR = 1.27; 95% CI: 1.07–1.52; *p = 0.007). The increase was 104% in regions with adequate access (IRR = 2.04) and 242% in regions with limited access (IRR = 3.42)."<br>We also note in the Discussion that this magnitude is both statistically and clinically meaningful, reflecting a short-term destabilization of FMF control.
4. Recall Bias: Since attack frequency was self-reported, recall bias should be more explicitly acknowledged. Patients with missing data on attack frequency were excluded, but the implications of this exclusion should be discussed.	We agree. Recall bias is now explicitly discussed in the Limitations: "Attack frequency data were obtained from patient diaries and physician-verified reports, which may still be susceptible to recall bias. Patients with missing monthly data were excluded to preserve data quality, but this may have led to underrepresentation of individuals with irregular follow-up."
5. Genetic Findings: Approximately 80% of patients had exon 10 MEFV mutations. Variant details should be provided (perhaps in a supplementary file) to support the pathogenicity of the variants.	We have expanded the genetic results and provided detailed MEFV variant information in the revised table. This includes the specific exon 10 mutations identified and their distribution, improving clarity regarding the effect of the detected variants.
6. Comorbidities: The most common comorbidity was juvenile idiopathic arthritis (JIA), followed by immunoglobulin A (IgA) vasculitis (Table 1). Percentages should be presented alongside actual numbers in the table for better interpretation.	We added.
7. Residence Data: The percentages of regions of residence presented in Table 1 are confusing and need clarification.	We have revised and clarified the residence data in Table 1, ensuring that percentages and categories are now presented in a clear and consistent manner.

8. Study Flow: The flow of patients is unclear. At the start, 1006 pediatric patients are mentioned, but 32 were excluded because they were adults. This creates confusion and should be clarified.	Thank you for this important observation. We initially mentioned the 1006 patients to explicitly show the number of excluded adult cases; however, we have now revised the text and flow description to clearly indicate that 963 pediatric patients were ultimately included. The study flow has been reorganized to avoid confusion
9. Figures and Tables:<br>o Figure 2: There are three lines, but color coding is given for only two. This should be corrected.	Corrected
o Supplementary Table 2: The main difference appears between patients in the earthquake area without resources and those outside the earthquake area or with sufficient resources. This suggests multiple reasons for increased episodes (e.g., medication issues). The handling of such confounding factors should be clarified.	Thank you for this insightful comment. Access to medication is ofcourse an important factor and we accept this in the paper. However, since medication access problems were confirmed to last less than two weeks in all affected patients, we believe that the long-term increase in attack frequency may be related to environmental factors and the persistent stress associated with the more severe destruction in the affected region, rather than to medication access issues, which would primarily explain only the first month.
o Definition of Limited Resources: The manuscript refers to “limited resources,” but this definition is missing. It should be explicitly stated.	“Earthquake area with limited/no drug access: earthquake-affected provinces where medication shortages or delivery problems for the first two weeks after the event, however, they got their colchicine after 2nd week.”
10. Interpretation of Results:<br>• Results should be presented with actual values before moving to interpretation.	We have provided all results in the section now.
• For example, the statement that “patients living in the earthquake zone experienced more frequent flares during the month of the earthquake and the following three months” is valuable, but precise numbers should be shown	To assess the temporal and regional changes in monthly attack counts before and after the earthquake, we fitted a multilevel interrupted time-series (ITS) and difference-in-differences (DiD) Poisson mixed-effects regression model. We modelled the probability of experiencing at least one attack in a given month (binary outcome: attack = 1 if ≥1 attack, else 0) between February 2022 and August 2023 to assess both immediate (level) and gradual (slope) post-earthquake effects across regions using a multilevel mixed-effects logistic regression. We revised results section.
• The conclusion that patients outside the directly affected area also had increased flares may suggest broader psychosocial or environmental stressors, which should be better substantiated.	This was indeed interesting adn we wanted to present this data for future observations. We think that this may be due to the vast media coverage of the sufferings, but we did not comment since this is our speculation only.

VERSION 2 – REVIEW

**REVIEWER NAME**	Flom, Peter
**REVIEWER AFFILIATION**	Peter Flom Consulting
**REVIEWER CONFLICT OF INTEREST**	No Conflict of Interest
**AI DISCLOSURE**	No, I have not used AI

**DATE REVIEW RETURNED**	14-Jan-2026

**GENERAL COMMENTS**	The authors have addressed my concerns and I now recommend publication
